# Glioblastoma with Both Oligodendroglioma and Primitive Neuroectodermal Tumor-Like Components in a Case with 9-Year Survival

**DOI:** 10.1155/2018/1382680

**Published:** 2018-06-11

**Authors:** Ying-Tso Chen, Shu-Shong Hsu, Chi-Man Yip, Ping-Hong Lai, Huai-Pao Lee

**Affiliations:** ^1^Division of Neurosurgery, Department of Surgery, Kaohsiung Veterans General Hospital, Kaohsiung, Taiwan; ^2^Department of Surgery, Kaohsiung Veterans General Hospital, Kaohsiung, Taiwan; ^3^Department of Surgery, National Defense Medical Center, Taiwan; ^4^Department of Radiology, Kaohsiung Veterans General Hospital, Kaohsiung, Taiwan; ^5^Department of Pathology, Kaohsiung Veterans General Hospital, Kaohsiung, Taiwan; ^6^Department of Nursing, Meiho University, Pingtung, Taiwan

## Abstract

**Introduction:**

Glioblastoma multiforme (GBM), the most common primary malignant brain tumor in adults, is characterized by extensive heterogeneity in its clinicopathological presentation. A primary brain tumor with both astrocytic differentiation and neuronal immunophenotype features is rare. Here, we report a long-term survival patient who presented this rare form of GBM in the disease course.

**Presentation of Case:**

A 23-year-old woman, presenting with rapidly progressive headache and right-side weakness, was diagnosed with brain tumor over the left basal ganglion. She underwent the first craniectomy for tumor removal, and histopathology revealed classic GBM. Tumor recurrence occurred 8 years later. Another gross total resection was performed and pathology revealed GBM with the oligodendroglioma component (GBM-O). Due to disease progression, she received debulking surgery the following year. The third pathology revealed glioblastoma with primitive neuroectodermal tumor-like component (GBM-PNET).

**Discussion:**

GBM-PNETs are collision tumors with both neuronal and glial components. They are rare, and a few case reports have suggested that these tumors are associated with favorable outcomes but a higher risk of cerebrospinal fluid dissemination.

**Conclusion:**

We report a patient who developed the distinct pathologic variants of classic GBM, GBM-O, and GBM-PNET, throughout the disease course. Young age, aggressive surgical resection, and pathologic and genetic features may have contributed to the long-term survival of the patient.

## 1. Introduction

Glioblastoma multiforme (GBM) is the most malignant form of astrocytoma and the most frequent primary tumor of the brain in adults. Its prognosis is poor, with a median survival of 12.1–14.6 months despite current multimodal treatment [1]. GBM shows a great degree of histological variability and numerous morphologic subtypes, some of which may be associated with specific genetic alterations or clinical behaviors [2]. Glioblastoma with primitive neuronal component, which has been referred to as glioblastoma with the primitive neuroectodermal tumor-like component (GBM-PNET) in the literature and which is one of the emerging GBM subtypes, is a rare tumor with the combined features of malignant glioma and PNET. Here, we report a long-term survival patient who presented not only this rare collision tumor but also different histopathological components in the disease course.

## 2. Case Presentation

**Figure 1:**
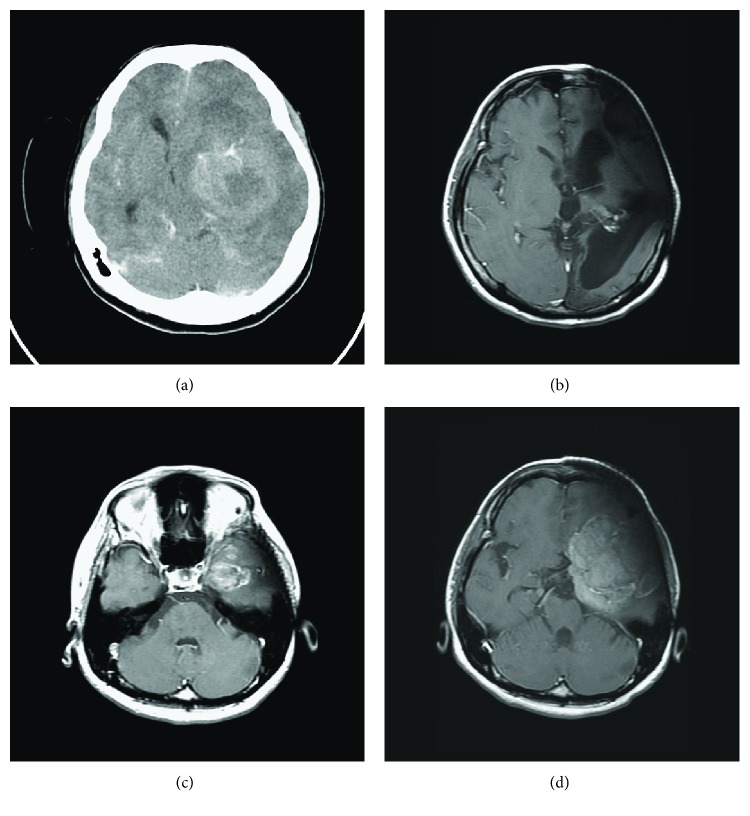
(a) Brain computed tomography in December 2006 showed an enhanced brain tumor over the left temporal region with mass effect; (b) no obvious recurrent tumor was found by brain MRI in 2013; (c) recurrent tumor was found by MRI in 2014, before the second surgery; and (d) brain MRI in May 2015, before the third surgery, revealed tumor progression.

A 23-year-old woman presented with progressive headache, nausea, and vomiting for 1 week. Right-side weakness, ptosis, and diplopia were also found. Due to acute onset conscious disturbance (Glasgow Coma Scale of E3VaM5) in the hospital, brain computed tomography was arranged and revealed an enhanced brain tumor with necrotic cystic change. This tumor was located at the left temporal lobe with upward extension to the left basal ganglion and periventricular region, causing perifocal edema and midline shift (Figure 1). We performed emergent craniectomy for tumor removal in December 2006.

**Figure 2:**
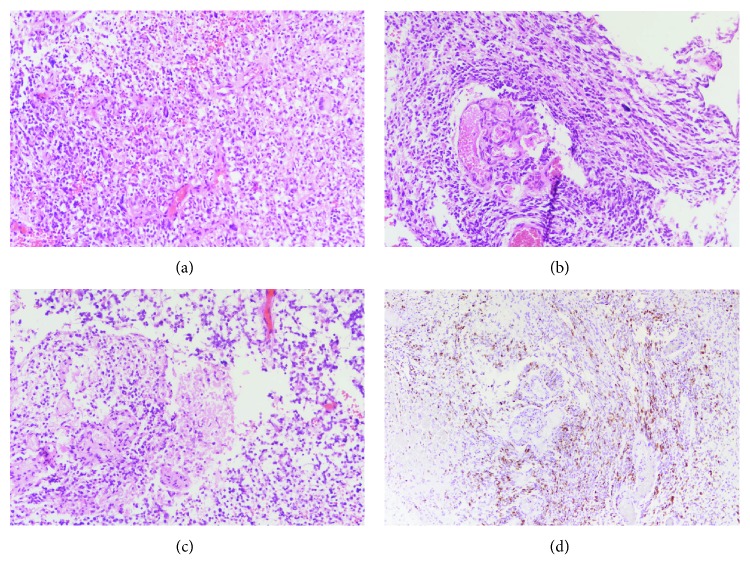
(a) Markedly increased cellularity of neoplastic astrocytes with nuclear pleomorphism and hyperchromasia (200x), (b) microvascular proliferation (200x), (c) necrosis (200x), and (d) increased proliferation index of tumor cells as revealed by Ki-67 immunostaining (16.3%) (100x).

Pathology revealed pleomorphic, hyperchromatic cells with glassy, astrocytic cytoplasm, as well as hypercellularity, microvascular proliferation, and necrosis, consistent with the diagnosis of classic GBM (Figure 2).

The patient underwent radiotherapy 1 month later and followed by chemotherapy with temozolomide for 6 months. Her performance status improved to a Karnofsky Grade of 70, and her clinical condition was stable thereafter. However, follow-up brain magnetic resonance imaging (MRI) in June 2014 revealed a new enhanced nodular lesion, approximately 1.1 cm in diameter, at the left temporal base. The brain MRI in October 2014 revealed a progressive change of lesions, maximum 3.0 cm in diameter (Figure 1). Thus, she again received surgery for gross tumor removal.

**Figure 3:**
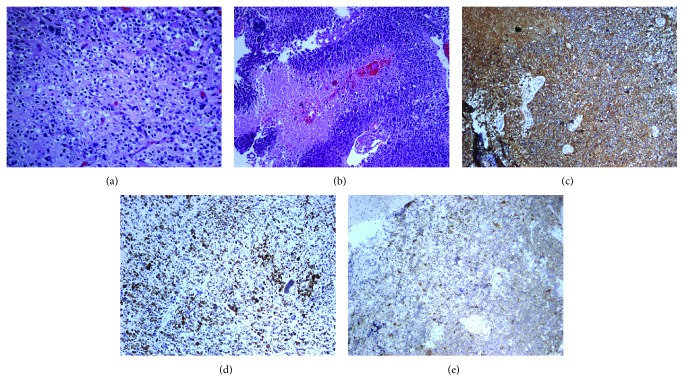
(a) Foci of oligodendroglial component featuring clear cytoplasm, round nuclei (200x). The neoplastic cells showed (b) necrosis (100x), (c) GFAP(+) (100x), (d) p53(+) (100x), and (e) IDH1(+) (100x).

Histologically, except for the necrosis feature of GBM, the tumors showed the oligodendroglial component. Neoplastic cells also showed isocitrate dehydrogenase 1(IDH1)(+), p53(diffuse +), and O^6^-methylguanine-DNA methyltransferase (MGMT)(−) as revealed by immunostaining (Figure 3).

**Figure 4:**
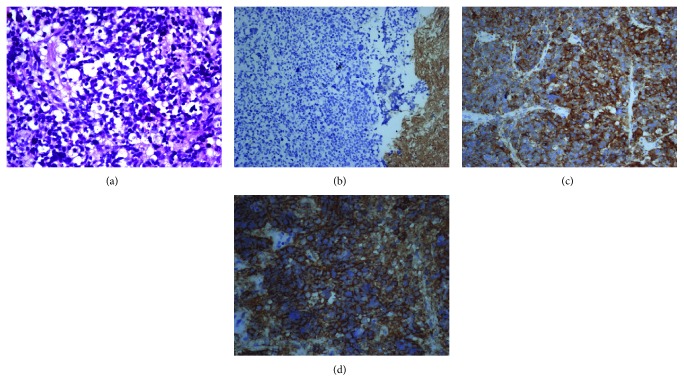
(a) Neoplastic cells with nuclear pleomorphism, hyperchromatism, abundant mitosis, and apoptosis (200x). Immunohistochemistry revealed (b) focal weak positivity for GFAP (200x), (c) synaptophysin(+) (400x), and (d) CD56(+) (400x).

During follow-up, signs of increased intracranial pressure were noted in May 2015. Therefore, she received a third debulking surgery. The third pathology revealed both GBM- and PNET-like components. In immunohistochemistry, the PNET-like component exhibited positivity for synaptophysin and CD56 and focal weak positivity for glial fibrillary acidic protein (GFAP) (Figure 4).

One month after the surgery, her condition rapidly deteriorated. The patient and her family chose to pursue hospice care. She passed away with a total 9-year survival since diagnosis (Table 1).

## 3. Discussion

According to the current 2016 WHO classification system, GBM can be divided into IDH wild type, IDH mutant, and NOS (not otherwise specified). Giant cell glioblastoma, gliosarcoma, and epithelioid glioblastoma are categorized under IDH wild-type glioblastoma [3]. Moreover, based on the described histopathology, diverse glioblastoma variants are still emerging, such as glioblastoma with primitive neuroectodermal tumor-like component (GBM-PNET), GBM with oligodendroglioma component (GBM-O), small cell astrocytoma, and granular cell astrocytoma [2]. Recent studies have suggested that under the stochastic or hierarchical hypothetical model, the presence of a cancer stem cell may account for the substantial diversity in the pathological characteristics of GBM [4].

GBM-Os account for 4% to 27% of all GBMs in previous studies. They feature both astrocytic and oligodendroglial differentiation, with the typical fried-egg appearance. Recent analyses of large tumor databases found a significantly higher IDH1 mutation rate in GBM-Os (31% versus <5%) [5]. Moreover, the EORTC 26981/NCICCE.3 trial found that GBM-Os showed significantly higher levels of IDH1 mutation (19% versus 3%) than GBMs [6]. Thus, GBM-Os exhibit a higher frequency of IDH1 mutations and a lower frequency of PTEN deletions, which is a characteristic distinct from that of classic GBMs. Recent studies have revealed that GBM-Os tend to occur at a younger age and that patients with GBM-Os exhibit longer survival than those with other forms of GBMs [7, 8].

In contrast to GBMs, those previously designated as PNETs, incorporated into the term embryonal tumor with multilayered rosettes (ETMR) in the 2016 WHO classification system for CNS tumors, are aggressive neoplasms, with medulloblastoma-like histology, and mostly affect the pediatric population. They are composed of poorly differentiated neuroepithelial cells and are associated with a high risk of metastases through cerebrospinal fluid dissemination. Patients with PNETs exhibit a poor prognosis, requiring craniospinal radiation in addition to platinum-based chemotherapy [3, 9]. GBM-PNETs are a rare pattern in GBMs. They contain the architectures of both traditional GBM and PNET-like areas, with hypercellularity, minimal fibrillary background, high nuclear-to-cytoplasmic ratio, oval-round hyperchromatic nuclei, high mitotic-karryorhectic indices, and Homer Wright neuroblastic rosettes. Furthermore, PNET-like areas show lower GFAP expression but positive staining for S-100, synaptophysin, NeuN, and neurofilament protein (NFP) [10–12].

One of the largest series on GBM-PNETs reported 53 cases, with a male : female ratio of 1.3, leptomeningeal metastasis in up to 40% of patients, and the median survival of 9.1 months [11]. However, recent studies have suggested favorable prognostic features for GBM-PNETs. A study of 40 cases of grade III or IV glioma with PNET components, as demonstrated by GFAP and NFP coexpression, reported a low recurrence rate (36%) and a mean survival of 44 months after gross total resection [12]. In another study of 12 patients with GBM-PNETs, IDH1 mutations were observed in 2 patients with an overall survival of 15 and 31 months. The present study also concluded that GBM-PNETs have a higher frequency of IDH1 mutation and might have favorable prognosis [13].

In our case, we observed different pathological variants of glioblastoma in the disease course. Both histopathologic types of GBM-Os and GBM-PNETs tend to contribute to a favorable prognosis. In addition, probably because of initial radical surgical resection, young age at the time of diagnosis, silencing of MGMT, and positive IDH-1 staining, the patient had long-term survival.

## 4. Conclusion

GBM is associated with substantial diversity in its clinicopathological characteristics. GBM-Os resemble GBMs but also contain areas resembling oligodendroglioma, whereas GBM-PNETs are rare tumors with the combined features of high-grade glioma and primitive neuroectodermal tumors. This report illustrates that the distinct histopathologic variants of glioblastoma can appear throughout the disease course and such tumors are associated with favorable outcomes.

## Figures and Tables

**Table 1:** Timeline.

Dates	Relevant past medical history and interventions		

December 2006	23 y/o female with progressive headache, nausea, and vomiting for one week		

Date	Summaries from initial and follow-up visits	Diagnostic testing (including dates)	Interventions

December 20, 2006	Right-side weakness, ptosis and diplopia, conscious drowsiness	Brain CT	Emergent craniectomy for removal of tumor
		Pathology: classic GBM	
January 18, 2007~March 07, 2007			RT
March 2007~January 2008			6 courses of Temadol
June 09, 2011			Cranioplasty
October 10, 2014		MRI—progressive change of several tumors over the left temporal base	
December 02, 2014			Craniotomy for tumor removal
		Pathology—glioblastoma multiforme, with oligodendroglial component	
May 07, 2015	Headache, nausea, and vomiting	MRI—tumor local recurrence, with ventricle seeding	
May 13, 2015			Craniotomy for tumor removal
		Pathology—glioblastoma multiforme with PNET-like component	
June 2015	Signs of increased intracranial pressure	Brain CT: tumor progression	Conservative treatment
July 16, 2015	Expired		
